# The Genomic Landscape of Divergence Across the Speciation Continuum in Island-Colonising Silvereyes (*Zosterops lateralis*)

**DOI:** 10.1534/g3.120.401352

**Published:** 2020-07-13

**Authors:** Ashley T. Sendell-Price, Kristen C. Ruegg, Eric C. Anderson, Claudio S. Quilodrán, Benjamin M. Van Doren, Vinh L. Underwood, Tim Coulson, Sonya M. Clegg

**Affiliations:** * Department of Zoology, University of Oxford, United Kingdom; † Center for Tropical Research, Institute of the Environment and Sustainability, University of California Los Angeles, California; ‡ Department of Biology, Colorado State University, Fort Collins, Colorado; § Fisheries Ecology Division, Southwest Fisheries Science Center, National Marine Fisheries Service, NOAA, Santa Cruz, California; ** Environmental Futures Research Institute, Griffith University, Queensland, Australia

**Keywords:** Genomic heterogeneity, Gene flow, Genomic islands, Genomic valleys, Simulated divergence, RAD sequencing, Genomic landscape

## Abstract

Inferring the evolutionary dynamics at play during the process of speciation by analyzing the genomic landscape of divergence is a major pursuit in population genomics. However, empirical assessments of genomic landscapes under varying evolutionary scenarios that are known *a priori* are few, thereby limiting our ability to achieve this goal. Here we combine RAD-sequencing and individual-based simulations to evaluate the genomic landscape of divergence in the silvereye (*Zosterops lateralis*). Using pairwise comparisons that differ in divergence timeframe and the presence or absence of gene flow, we document how genomic patterns accumulate along the speciation continuum. In contrast to previous predictions, our results provide limited support for the idea that divergence accumulates around loci under divergent selection or that genomic islands widen with time. While a small number of genomic islands were found in populations diverging with and without gene flow, in few cases were SNPs putatively under selection tightly associated with genomic islands. The transition from localized to genome-wide levels of divergence was captured using individual-based simulations that considered only neutral processes. Our results challenge the ubiquity of existing verbal models that explain the accumulation of genomic differences across the speciation continuum and instead support the idea that divergence both within and outside of genomic islands is important during the speciation process.

Darwin described the process of speciation as a continuum in which differentiation between populations accumulates, first giving rise to well-marked ‘varieties’ or ‘races,’ and in some instances culminating in the formation of new species (Darwin 1859), a view that today is widely accepted (Mallet 2008; Nosil 2012; Powell *et al.* 2013). Numerous evolutionary processes can promote, stall or reverse trajectory along the speciation continuum, including gene flow, selection, recombination, mutation and drift (Ricklefs and Bermingham 2007; Ravinet *et al.* 2017; Kearns *et al.* 2018). Understanding how these processes interact at different stages and how this interaction shapes the speciation process remains a central but challenging goal in evolutionary biology (Marie Curie SPECIATION Network *et al.* 2012; Ravinet *et al.* 2017). Examining divergence at the level of the genome is a potentially powerful way to address this challenge (Nosil and Feder 2012; Seehausen *et al.* 2014).

During the divergence process, some regions of the genome diverge rapidly and others more slowly (Seehausen *et al.* 2014). This variation in the tempo of divergence across the genome results in a heterogeneous genomic landscape (Nosil *et al.* 2009; Andrew and Rieseberg 2013; Feder *et al.* 2013; Seehausen *et al.* 2014; Feulner *et al.* 2015; Ravinet *et al.* 2017). During the initial stages of the speciation continuum, divergence is expected to be localized to regions of the genome where loci are under strong divergent selection, forming peaks of divergence often referred to as ‘genomic islands of divergence’ (Feder *et al.* 2013; Seehausen *et al.* 2014). As populations move along the speciation continuum, genomic islands are predicted to widen as linkage disequilibrium facilitates divergence of neutral and weakly selected loci via divergence hitchhiking (Nosil *et al.* 2009; Feder and Nosil 2010). The growth of genomic islands of divergence forms the basis of the divergence hitchhiking model of speciation (Via 2012), which until recently was the prevailing mechanism through which genome-wide levels of divergence were thought to be achieved (Smadja *et al.* 2008; Via and West 2008). However, the conditions under which divergence hitchhiking can generate large regions of differentiation are limited, requiring small effective population sizes, low rates of migration, and strong selection (Feder and Nosil 2010).

Using closely related taxa at different stages of divergence as a proxy for the speciation continuum, a number of studies have provided evidence that the pattern of genomic divergence accumulates in a way consistent with the mechanisms described above (Martin *et al.* 2013; Feulner *et al.* 2015; Supple *et al.* 2015; Vijay *et al.* 2016). However, the pace at which divergence accumulates could be accelerated or hindered by other evolutionary processes. For example, gene flow could slow the rate at which divergence accumulates by having a homogenizing effect at neutrally evolving or weakly selected loci (Nosil *et al.* 2017). In contrast, in genetic isolation, divergence proceeds via selection and/or drift, unfettered by this homogenizing effect. As such, these different modes of divergence are expected to affect the distribution of genetic divergence values in predictable ways, accounting for the stage of the speciation continuum (Feder *et al.* 2012) (see Figure 1). To date, comparisons of genomic divergence between races of *Heliconius* butterflies distributed in allopatry *vs.* those in parapatry (Martin *et al.* 2013) provide the strongest empirical basis for the likely role of gene flow in shaping the genomic landscape. However, studies are needed with comparisons matched for divergence timeframe and with rates of gene flow inferred from more than geographic distribution alone.

**Figure 1:**
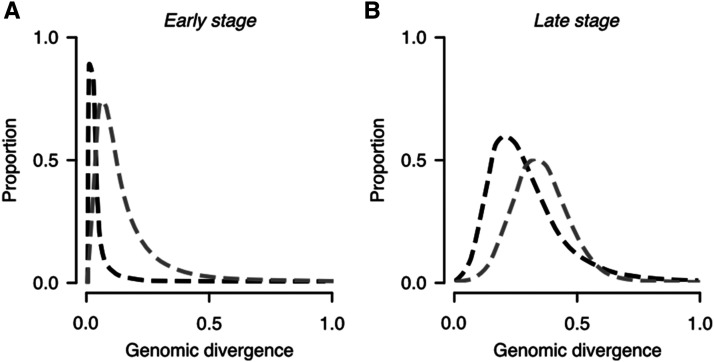
Hypothetical distributions of genetic differentiation expected for divergence with and without gene flow. (A) During the early stages of the speciation continuum divergence is expected to be limited to few loci, resulting in a highly skewed distribution of divergence values. This expectation will be most extreme for populations diverging with gene flow (black lines) than for populations diverging in genetic isolation (gray lines) as the homogenizing effect of gene flow limits divergence to only loci under strong divergent selection. (B) During later stages of the speciation continuum skew is expected to break down as divergence accumulates, but less so in populations diverging with gene flow (black lines) than those diverging without (gray lines) as occasional gene flow may reduce levels of divergence at positions of the genome even as reproductive isolation is approached.

Genomic valleys – highly conserved regions where differentiation falls far below background levels – are also a common feature of the genomic landscape. While these have been identified in a range of species (Hofer *et al.* 2012; Roesti *et al.* 2012; Wang *et al.* 2016; Van Doren *et al.* 2017), their role in shaping the genomic landscape has been overlooked in comparison to the focus on genomic islands. Genomic valleys may play an important role in maintaining genomic heterogeneity by slowing the approach to genome-wide divergence. Genomic valleys may occur because of alleles favored in both populations (parallel adaptation) (Nielsen 2005; Roesti *et al.* 2012), or they may result from purifying selection in which newly arising deleterious mutations are selected against in order to preserve biological function (Cvijović *et al.* 2018). Alternatively, genomic valleys may occur due to incomplete lineage sorting at neutral loci, where diverging populations share alleles for some time, manifesting as regions of below background level divergence (Stölting *et al.* 2013). Should parallel adaptation be important in their formation, we would expect the position of genomic valleys to closely correspond to the position of loci under parallel selection. Further, as parallel selection leaves a distinct signature of reduced diversity, where genomic islands are the product of parallel selection, between population diversity (*d*_xy_) would be expected to be reduced (Roesti *et al.* 2014). A similar pattern of reduced *F*_st_ and reduced *d*_xy_ is expected under purifying selection (Cvijović *et al.* 2018). Where genomic valleys are the product of incomplete lineage sorting at neutral loci, they are expected to be more numerous during the early stage of divergence as shared alleles become less numerous over time. Under these proposed mechanisms, a reasonable expectation of the temporal dynamics of genomic valleys is that they decrease in size over longer divergence timeframes, as genomic valleys are broken down by recombination and loosely linked/neutral loci diverge (Charlesworth 2006). This expectation is yet to be tested empirically.

A more nuanced understanding of how genome-wide divergence develops from the heterogeneous genomic landscape requires simultaneous consideration of the stage along the speciation continuum and the gene flow context of divergence for systems that have well characterized population histories. Members of the silvereye subspecies complex (*Zosterops lateralis*) of Australia and southwest Pacific islands offer an exceptional opportunity to explore patterns of divergence across different timescales and gene flow contexts. The species has repeatedly colonized islands in the region since its origin on the Australian mainland (Mees 1969) across timeframes from decades to hundreds of thousands of years. This provides a spectrum of divergence timeframes that can be used as a proxy for the speciation continuum, capturing incipient phenotypic divergence through to highly divergent subspecies (Clegg *et al.* 2002a). Importantly, silvereye populations have diverged under different gene flow scenarios, with some populations diverging with gene flow and others diverging in genetic isolation (Mees 1969). Silvereyes also show a repeated pattern of phenotypic change on islands toward increased body and bill size (Mees 1969; Clegg *et al.* 2002b; Clegg *et al.* 2008). While we do not have direct evidence that body size acts as a reproductively isolating mechanism, empirical and modeling evidence supports that increased size is the result of directional natural selection that is strongest in the early generations following colonization of novel environments (Clegg *et al.* 2002b; Clegg *et al.* 2008). There are several hypotheses to explain the selective advantage of larger body size, including the positive relationship between successful intraspecific aggressive encounters and body size in high density insular populations (Robinson‐Wolrath and Owens 2003), and competitive displacement in the presence of closely related species. While we do not know the genetic architecture of this repeated phenotypic pattern, several studies have found genes of large effect for body/bill traits (Bosse *et al.* 2017; Chaves *et al.* 2016; Cornetti *et al.* 2015; Lamichhaney *et al.* 2015; vonHoldt *et al.* 2018) and we anticipate that genomic islands should form around loci underlying this phenotypic change across comparisons, allowing the dynamics of individual genomic islands to be studied.

Here we use empirical analysis of Restriction-site Associated DNA Sequencing (RAD-Seq) data from silvereyes and individual-based simulation models to address the following questions: 1) How does genomic divergence accumulate across the genome over time? 2) How is the accumulation of divergence affected by gene flow? 3) Does the position of genomic islands of divergence and valleys of similarity correspond to the location of loci under divergent selection or parallel/purifying selection, respectively? 4) Do genomic islands widen, and valleys narrow, over longer divergence timeframes? and 5) Are the features of genomic valleys consistent with proposed mechanisms that could contribute to their formation?

## Materials & Methods

### Sample collection

**Figure 2:**
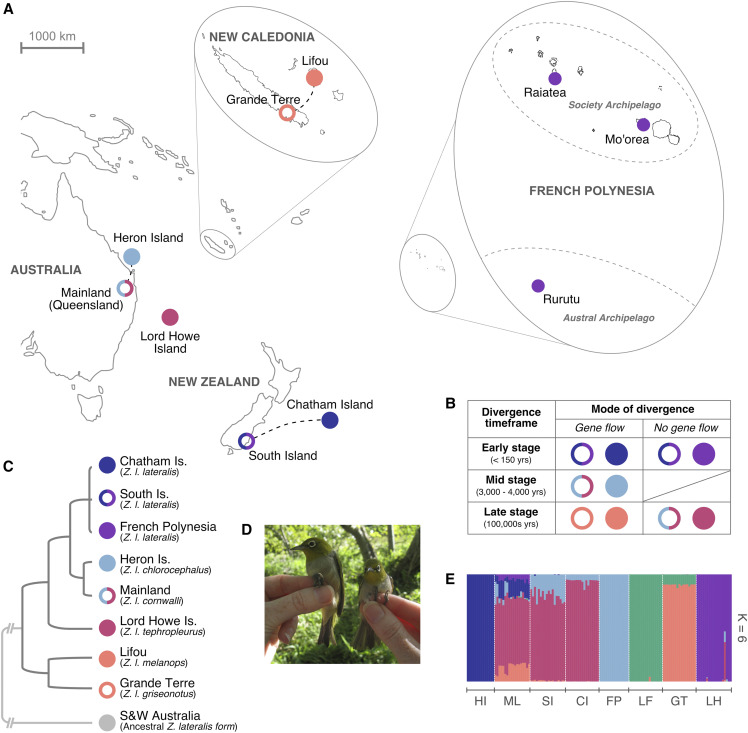
Silvereye populations used in the study. (A) Sampling locations of *Zosterops lateralis* across the south Pacific (dashed lines indicate gene-flow between populations); (B) Population divergence timeframes (known from historical records, or inferred from island ages and genetic divergence dates) and modes of divergence (gene-flow or no gene-flow); (C) evolutionary relationships between *Z. lateralis* subspecies and populations based on mitochondrial DNA (Black, R., unpublished results), SNP data (Sendell-Price, A.T., unpublished results) and known colonization histories (subspecies indicated in brackets); (D) *Z. l. chlorocephalus* from Heron Island (left) are up to 40% larger than *Z. l. cornwalli* from the Australian mainland (right). Image by Nick Clark; (E) Maximum likelihood estimation of individual ancestries calculated with ADMIXTURE (Alexander *et al.* 2009), based on 6,822 LD-filtered SNPs.

Blood samples were collected from eight silvereye populations across the south Pacific, capturing a range of divergence levels and gene flow variation within this species, and providing five diverging population pairs for comparison. Population pairs varied in their divergence timeframes (early stage: <150 years, mid stage: 3,000-4,000 years, and late stage: 100,000s years) and mode of divergence (gene flow or no gene flow) (Figure 2). The populations used in this study provide a rare example of where gene flow scenarios and divergence timescales are either known *a priori* or where a strong case can be made to infer the likely gene flow scenario. This is based on a combination of historical records, contemporary bird movements, geographic proximity, and phylogenetic inferences as outlined below:

#### South Island *vs.* Chatham Island:

The Chatham Island silvereye population was colonized from South Island (New Zealand) during the 1850s. This timing is historically documented (Mees 1969; Clegg *et al.* 2002a) and reflected in population level phylogenies (R. Black, unpublished results). In the mid 1800s, South Island silvereyes were undergoing a population expansion which resulted in the colonization of other islands in the region (notably: North Island of New Zealand and Norfolk Island) (Mees 1969; Clegg *et al.* 2002a). Given that population expansion was taking place within this region for several decades after the colonization of Chatham Island, these population are likely to have experienced gene flow. Therefore, this comparison is characterized as Early Stage – Gene Flow.

#### South Island *vs.* French Polynesia:

The French Polynesia silvereye population is the product of a documented human-mediated introduction of South Island silvereyes (*Z. l. lateralis*) to Tahiti in 1937 (Monnet *et al.* 1993; Thibault and Cibois 2017). Given that French Polynesia is located well beyond the natural distribution limit for this species and that the French Polynesian population is thought to be the product of a single and recent introduction event (Sendell-Price *et al.* 2020), this comparison is characterized as Early Stage – No Gene Flow.

#### Mainland (Australia) *vs.* Heron Island:

The Heron Island silvereye population was established from the mainland silvereye subspecies (*Z. l. cornwalli*) between 3,000-4,000 years ago. This divergence timeframe is supported by geological records which suggest that Heron Island has been vegetated (and therefore habitable) for a maximum of 4,000 years (Degnan and Moritz 1992; Hopley 1982). Given that *Z. l. cornwalli* is regularly observed on Heron Island, this comparison is characterized as Mid Stage – Gene flow.

#### Grande Terre *vs.* Lifou:

Based on mitochondrial DNA divergence estimates, silvereyes on the islands of Grande Terre (*Z. l. griseonotus*) and Lifou (*Z. l. melanops*) diverged in the past few hundred-thousand years (Black, R., unpublished results). Given the silvereye’s ability to cross water boundaries and the geographic proximity of these islands (200km apart), this comparison is characterized as Late Stage – Gene Flow.

#### Mainland (Queensland) *vs.* Lord Howe Island:

Based on phylogenetic analyses, *Z. l. tephropleurus* on Lord Howe Island is derived from the mainland subspecies *Z. l. cornwalli* in the past few hundred-thousand years (A. T. Sendell-Price, unpublished results). Given the geographic isolation of Lord Howe Island, this comparison is characterized as Late Stage – No Gene Flow.

For each of the population comparisons used in this study the diverging populations show body size differentiation. *Z. l. lateralis* from Chatham Island and French Polynesia are larger than those from their South Island source (Clegg *et al.* 2002a; Sendell-Price *et al.* 2020), *Z. l. chlorocephalus* from Heron Island is approximately 40% larger than its mainland ancestor Z. *l. cornwalli* (Clegg *et al.* 2008), *Z. l. melanops* from Lifou is larger in many traits than its Grand Terre ancestor, *Z. l. griseonotus* (Black, R., unpublished results) and *Z. l. tephropleurus* from Lord Howe Island is substantially larger than its mainland ancestor (Clegg *et al.* 2002a; Clegg and Phillimore 2010).

Sampling locations and collection dates are provided in Table 1. To provide large enough sample sizes for analysis, the French Polynesian population included samples taken from multiple islands. This is justified based on an admixture analysis which grouped these samples together into a single cluster (Figure 2E). All birds were caught using mist nets or traps and 20–40 μl of blood collected from the brachial wing vein was stored in ∼0.5 ml of lysis buffer (0.01M Tris-HCl; 0.01M NaCl; 0.01M EDTA; 1% n-lauroylsarcosine, pH 8.0) (Seutin *et al.* 1991).

**Table 1:** *Z. lateralis* sampling information. No. Sequenced = number of individuals including in RAD-Seq libraries; No. Filtered = number of individuals retained after quality filtering

Subspecies	Pop. code	Sampling location	Lat / Long	Collection date	No. Sequenced	No. Filtered
*chlorocephalus*	HI	Heron Island	−23.44 / 151.91	2015	20	14
*cornwalli*	ML	Mainland Australia	−28.18 / 153.45	2013	20	18
*griseonotus*	GT	Grande Terre	−21.78 / 166.02	2014	20	17
*lateralis*	CI	Chatham Island	−44.58 / 176.32	1997	22	17
*lateralis*	SI	South Island	−45.53 / 170.30	1997	23	18
*lateralis*	FP	Mo’orea	−17.51 / -149.91	2005	17	10
*lateralis*	FP	Raiatea	−16.82 / -151.46	2010	3	2
*lateralis*	FP	Rurutu	−22.48 / -151.34	2012	3	3
*melanops*	LF	Lifou	−20.89 / 167.25	2014	20	17
*tephropleurus*	LH	Lord Howe Island	−31.33 / 159.05	1998	20	18

### RAD-PE sequencing and bioinformatics

Restriction-site associated DNA paired-end (RAD-PE) sequencing was used to assess the genomic landscape of divergence among silvereye populations. This reduced representation sequencing method has the potential to identify many hundreds of thousands of Single Nucleotide Polymorphisms (SNPs) distributed throughout the genome (Davey and Blaxter 2010). Genomic DNA in blood samples was extracted from between 20 and 23 individuals per population (or set of populations in the case of French Polynesia) (n = 168) using QIAGEN DNeasy blood and tissue extraction kits following manufacturer protocols. RAD-PE libraries were then prepared using restriction enzyme *SbfI*-HF (New-England Biolabs Inc., Beverly MA, USA) following the protocol by Ali *et al.* (2016). See supplementary material for a comprehensive description of library preparation. Libraries were sequenced on three Illumina HiSeq4000 lanes (Illumina, San Diego, CA, USA) at the UC Davis Genome Center using paired-end 150-bp sequence reads.

Quality of sequencing reads was checked visually using FASTQC (Available online at: http://www.bioinformatics.babraham.ac.uk/projects/fastqc). The *process_radtags* script included in the STACKS version 1.4 software pipeline (Catchen *et al.* 2013) was used to assign sequence reads to individuals. In addition, reads containing uncalled bases and/or bases of low quality were discarded in this step using default quality thresholds (an average Phred score of 10 in sliding windows of 15% of the length of the read). Sequences with possible adapter contamination and/or missing the *SbfI*-HF restriction site were also discarded. Following this, reads were filtered for PCR duplicates using the STACKS *clone_filter* script. The remaining reads were then mapped to the *Zosterops lateralis melanops* genome assembly version 1 (NCBI Assembly GCA001281735.1, (Cornetti *et al.* 2015)) with BOWTIE2 version 2.2.6 (Langmead and Salzberg 2012) using end-to-end alignment and default settings (allowing for a maximum of two mismatches in the seed (-n 2)). Genotypes were then called using the *HaplotypeCaller* and *GenotypeGVCFs* tools from the Genome Analysis Toolkit (GATK) nightly build version 2016-12-05-ga159770 (McKenna *et al.* 2010). To output both variant and non-variant sites, *HaplotypeCaller* was ran using -output_mode ’EMIT_ALL_CONFIDENT_SITES’ and *GenotypeGVCFs* ran using mode ‘–includeNonVariantSites’. The resulting output was then filtered to remove indels and only include sites where the minor allele count was ≤ 2; minimum genotype quality = 30; minimum depth = 8; and sites were called in at least 70% of individuals. Following this the outputted VCF file was further filtered to remove individuals missing > 30% of sites.

As the *Z. l. melanops* genome is only assembled to the scaffold level (Cornetti *et al.* 2015), *Z. l. melanops* scaffolds were mapped to chromosomes of the *Taeniopygia guttata* genome assembly version 3.2.4 (NCBI Assembly GCA_000151805.2) using Satsuma Synteny (Grabherr *et al.* 2010). Output from Satsuma Synteny was then used to assign scaffolds to chromosomes and determine order, location, and orientation using custom R scripts from Van Doren *et al.* (2017). Custom scripts from Sendell-Price *et al.* (2020) were then used to reorder the GATK outputted VCF file accordingly and remove SNPs where chromosomal positions could not be determined. Because synteny is high in birds (Ellegren 2010), 96.8% of the *Zosterops* scaffolds were assigned to assembled chromosomes and arranged in the presumed correct order and orientation. As inversions and other chromosomal rearrangements are known to occur in birds (Backström *et al.* 2010), it may be possible that a small percentage of scaffolds were ordered or oriented incorrectly. However, a small number of misplaced scaffolds is not expected to impact our overall interpretation of empirical patterns.

Finally, Wright’s (1951) fixation index (*F*_ST_ – a measure of relative divergence) and Nei’s (1987) measure of absolute divergence (*d*_xy_) were calculated in non-overlapping windows of 5kb, 50kb and 100kb for each population comparison, using Python scripts developed by Martin *et al.* (2013) (https://github.com/simonhmartin/genomics_general). *F*_ST_ and *d*_xy_ were outputted only for windows that contained at least 10 sites. For all downstream analyses that used windowed statistics we filtered our dataset so that windows containing missing data were removed, *i.e.*, only those windows containing *F*_ST_/*d*_xy_ for all pairwise comparisons were retained.

### Population genomic analyses

We assessed the shift from localized divergence at few loci toward more genome-wide levels of divergence by comparing the third moment (skewness) of *F*_ST_ distributions between population comparisons. In line with expectations outlined in Figure 1, skewness provides a useful metric to describe a population comparisons’ stage of transition from localized (high positive skew) to genome-wide levels of divergence (less positive skew). Note: as *F*_ST_ is bounded between 0 and 1, distributions are expected to become negatively skewed when populations have been diverging over very long timescales as most loci approach fixation. We tested for significant differences in the skewness of empirical distributions using a randomization test. For each pairwise comparison of diverging populations (*e.g.*, South Island *vs.* Chatham Island compared to South Island *vs.* French Polynesia) we first calculated a test statistic (the absolute difference in distributional skew of observed *F*_ST_ values) and compared this to test statistics calculated for 10,000 randomized distributions. Randomized distributions were produced by assigning observed *F*_ST_ values to comparisons at random without replacement. *P*-values were then calculated as the percentile of the distribution of randomized test statistics that the observed test statistic lied on.

R scripts, based on those used in Van Doren *et al.* (2017), were used to identify highly diverged regions (genomic islands of divergence) and regions of low divergence (genomic valleys of similarity) occurring across the genomic landscapes of the diverging populations. First, for each population comparison, a kernel-based smoothing algorithm was applied to windowed *F*_ST_ values (box density with bandwidth of 20), and the smoothed line compared to 10,000 smoothed lines obtained after permuting the order of the windows (see Ruegg *et al.* (2014)). Genomic islands were identified as any location where the observed smoothed line was greater than the most extreme value from the permutation distribution (see Van Doren *et al.* (2017)). In contrast, genomic valleys were identified as any region where the observed smoothed line was lower than the lowest value of the permuted distribution. We merged outlier regions separated by ten windows or fewer. For each genomic island and genomic valley, we calculated mean *F*_ST_ and *d*_xy_. Wilcoxon signed-rank tests were used to determine if *d*_xy_ within genomic islands and genomic valleys differed significantly from chromosomal background levels. To assess the effect of time and gene flow on the development of patterns of heterogeneity the number and size of identified genomic islands and valleys were compared across timescales and gene flow contexts using Kruskal-Wallis tests. In the main text we present results of island/valley detection using 50kb windows, however island/valley detection was also conducted using 5kb and 100kb windows – these results are presented in the supplementary material.

### Identifying outlier SNPs under selection

To identify genomic regions that may be under selection, we scanned for outlier loci using *PCAdapt*, a principal components-based method of outlier detection with a low rate of false-positive detection (Luu *et al.* 2017). *PCAdapt* requires selection of K principal components, based on inspection of a scree plot, in which K is the number of PCs with eigenvalues that depart from a straight line. *PCAdapt* then computes a test statistic based on Mahalanobis distance and controls for inflation of test statistics and false discovery rate (FDR). Outlier SNPs were identified using the following settings for all population comparisons: K = 2, MinMAF= 0.2, and FDR = 0.01. As *PCAdapt* has been shown to be sensitive to linkage-disequilibrium (LD) (Lotterhos *et al.* 2019), prior to conducting outlier detection we filtered SNPs so that those with very high LD (*r^2^* > 0.8) were removed. LD-filtering was conducted using the ‘--indep-pairwise 1000 kb 1 0.8’ command in PLINK (Purcell *et al.* 2007).

### Candidate gene analysis

To determine if genomic islands of divergence occurred around genes known to be associated with body and beak size differences in birds, we compiled a list of candidate genes based upon a literature review of gene expression and association studies. The resulting candidate gene list was restricted to only those genes where (1) locations within the zebra finch genome are known; and (2) genes were mapped to directly by SNPs sequenced as part of this study or genes were located within the 50kb windows used when summarizing divergence statistics. Positions of candidate genes were then compared to the position of identified genomic islands of divergence. The position of candidate genes was also compared to the position of outlier SNPs. Genes were determined to fall within genomic islands, if island bounds (start and end positions) overlapped gene start and end positions (this includes 5′ UTRs and introns). Likewise, outlier SNPs were determined as within candidate genes if they fell within 10kb of the gene start/end positions.

### Simulations of divergence

We performed individual-based simulations where genomic divergence could only occur via neutral processes and compared model predictions with empirically observed genomic patterns. The simulations consequently provide a neutral model. If such a model can generate patterns observed in nature, then it is not necessary to invoke selection as the underlying driver of observed patterns. In other words, the simulation modeling allowed us to address whether the transition from localized to more genome-wide divergence could be explained by drift alone, and also allowed us to explore the fine scale effects of gene flow and recombination by simulating divergence under various levels of gene flow and recombination (both of which were not possible with empirical comparisons alone).

The simulations of divergence are based on the framework available in the *R* package “glads” (genomic landscape of divergence simulations) (Quilodrán *et al.* 2020). This package allows individual-based simulation for demographic, genetic, and genomic data forward in time. The model considers two populations that may or may not evolve independently according to different levels of migration between them, allowing a level of gene flow. There are three hierarchical levels: genotypes, phenotypes and demographic rates. All individuals are characterized by sex and genetic identity.

Each population started with individuals with randomly drawn genotypes across 46,853 biallelic SNPs on chromosome 5. Each genotype was determined by sampling with replacement from the distribution of all observed genotyped birds at each SNP. Individuals were then assigned a sex assuming a 50% sex ratio and assigned to a population to produce two populations, each with an initial population size of 400 individuals (similar to the Heron Island breeding population size (Kikkawa and Wilson 1983)).

Each population was iterated forward on a per-generation time step. At each generation, individuals were assigned a number of offspring independent of genotype. Fitness (the number of offspring) was determined by randomly drawing from a Poisson distribution with a mean (λ) that varied with population density (*N*). We thus defined mean population fitness as the average number of offspring produced. We included density-dependence (*σ_dem_*) in this function to avoid exponential growth. The final fitness of individuals at a given time was computed as *Poisson* (*λ*) - *Nσ_dem_*. The density dependence (*σ_dem_*) kept the population within empirically observed bounds for the Heron Island population (100-300 breeding pairs, see Table S1). The mean population fitness (λ) was obtained by multiplying the average number of fledglings of observed clutches by the mean survival during the summer, autumn and winter of young individuals along with the generation time (Table S1). These parameter values were obtained from literature (Kikkawa and Wilson 1983, Brook and Kikkawa 1998, Clegg *et al.* 2002b, Sandvig *et al.* 2017). Male fitness was then scaled such that the total sum of male fitness equalled the total sum of female fitness. Mating pairs were formed by combining male and female parents as a function of their ranked fitness values. Because the fitness value of individuals is obtained from a random distribution, this process is equivalent to a random mating pair within populations. This approach assumes monogamous reproduction which is empirically observed (Robertson *et al.* 2001).

To determine offspring genotype, we first identified crossover points along the parental genomes by drawing random values via a Poisson process. This procedure considers the physical position of each SNP in the chromosome, within which crossovers are obtained from a Poisson distribution along the length of the chromosome. Recombination is thus a homogeneous Poisson process where the rate is defined by the expected per base pair recombination rate (*e.g.*, 1e‐08, corresponding to 1 cM/Mb). Four different recombination rates were simulated: 2 cM/Mb, 3 cM/Mb, 6 cM/Mb, 10 cM/Mb and 19 cM/Mb (translated to crossovers per base pair in our simulations). This corresponds to the range of values known from the collared flycatcher (*Ficedula albicollis*) genome (Kawakami *et al.* 2014) (Table S1). Flycatcher recombination rates were used, as unlike the zebra finch genome, the flycatcher is not thought to contain large recombination deserts (Backström *et al.* 2010). Copies of chromosome 5 were then segregated from the parental genomes with recombination occurring at each crossover. Migration rate (*m*) was set to zero in simulations of no-gene-flow scenarios. In divergence-with-gene-flow simulations, offspring either stayed in the same population or dispersed, with simulations repeated for values of *m* of 0.0001, 0.001 and 0.01. Simulations were run for a maximum of 2,000 generations (assuming a generation time of three years (Clegg *et al.* 2008)). More information about the individual based framework is available in Quilodrán *et al.* (2020). R code is available at GitHub: https://github.com/eriqande/glads.

### Data availability

Raw sequencing reads have been submitted to the National Center for Biotechnology Information (NCBI; https://www.ncbi.nlm.nih.gov/) under accession number PRJNA489169. Supplementary material, VCF files and custom scripts required to replicate analyses are available at figshare: https://doi.org/10.25387/g3.12482000.

## Results

### RAD-PE sequencing and bioinformatics

Overall, RAD-PE sequencing resulted in 1,149,472,877 paired-end reads. Post-quality filtering (removal of indels and only including sites where: the minor allele count was ≤2; minimum genotype quality = 30; minimum depth = 8; and genotypes were called in at least 70% of individuals) reads covered a total of 15,872,783 sites (of which 788,169 were biallelic). Of the 168 samples sequenced, 134 were retained after removing individuals where ≥30% of data were missing. The number of individuals retained per location ranged from 15 to 18 (Table 1).

### Overall levels of divergence

**Figure 3:**
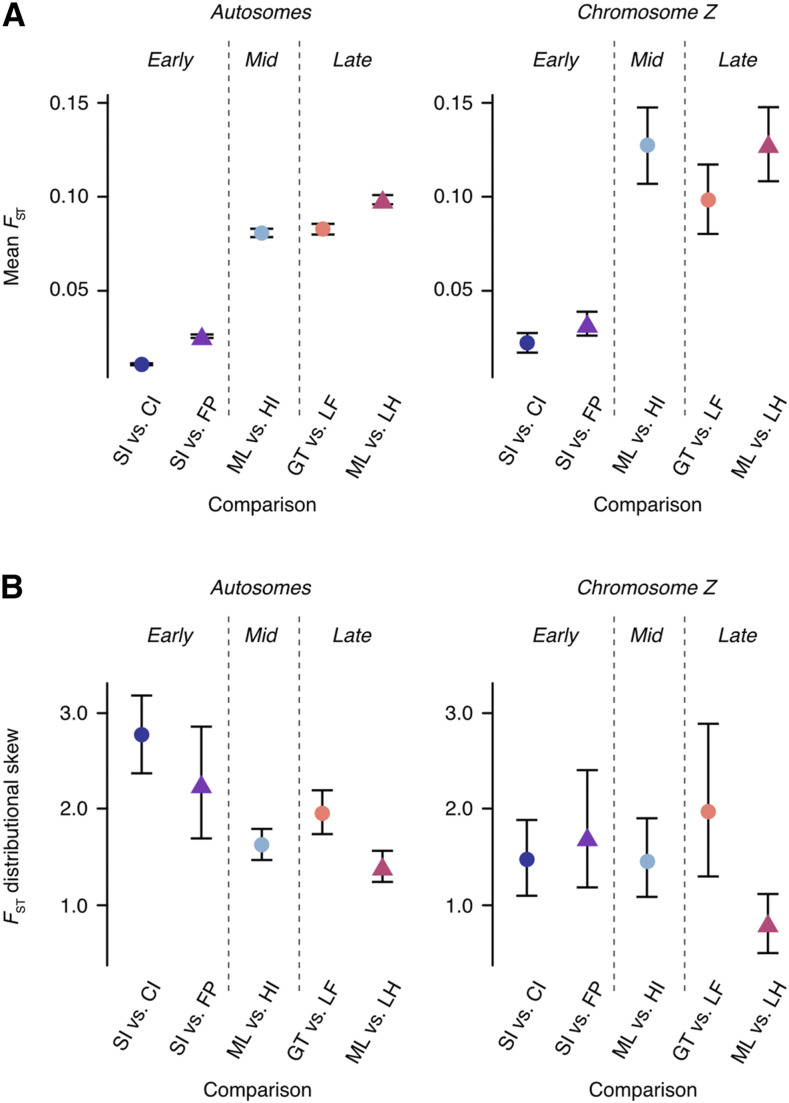
Mean *F*_ST_ and distributional skew of *F*_ST_ values. (A) Mean *F*_ST_ and (B) distributional skew of *F*_ST_ values calculated in 50kb non-overlapping windows. Calculated for autosomes and chromosome Z separately for each population comparison. Populations diverging with gene flow are indicated by circles and populations diverging in isolation by triangles. 95% confidence intervals obtained via bootstrapping over 500kb windows.

Mean *F*_ST_ was lowest for populations in the earliest stages of divergence (South Island *vs.* Chatham Island and South Island *vs.* French Polynesia) and increased over longer divergence timeframes, with populations at later stages of divergence (Grande Terre *vs.* Lifou and Mainland *vs.* Lord Howe Island) having the highest mean *F*_ST_ (Figure 3A). Mean *F*_ST_ was also consistently lower for populations diverging with gene flow when compared to non-gene flow scenarios matched for divergence timeframe (*i.e.*, South Island *vs.* Chatham Island compared to South Island *vs.* French Polynesia) demonstrating how the homogenizing nature of gene flow impedes divergence. Compared to autosomes, chromosome Z exhibited higher levels of divergence for all population comparisons (Figure 3A). This difference was significant for South Island *vs.* Chatham Island and Mainland *vs.* Heron Island comparisons (Mann-Whitney: all *P* values <0.05).

### Accumulation of genomic divergence across the genome

**Figure 4:**
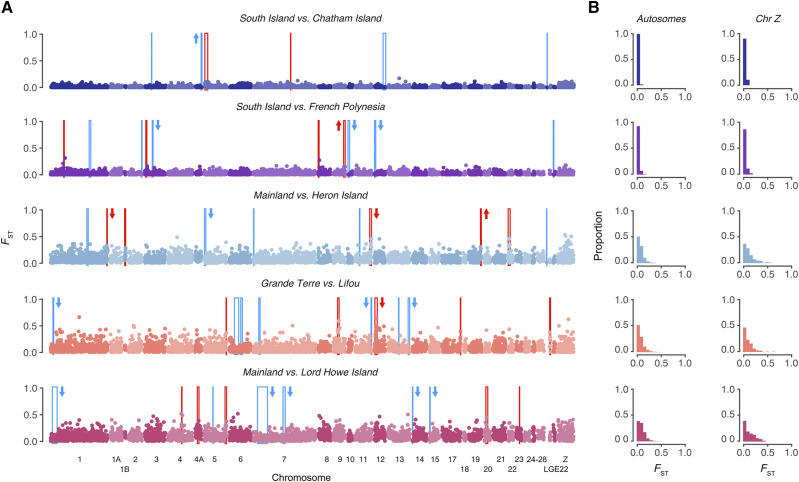
Distributions of divergence across the genome for diverging silvereye populations. (A) Pairwise *F*_ST_ across the genome for each population comparison calculated in non-overlapping 50kb windows. Regions of elevated differentiation (genomic islands) are highlighted in red and regions with low differentiation (genomic valleys) are highlighted in blue. Arrows beside genomic islands/valleys indicate if *d*_xy_ within these regions was significantly elevated (upwards pointing arrows) or decreased (downward pointing arrows) compared to chromosomal background levels. Significance determined using Wilcoxon signed-rank tests. Chromosomes are numbered according to the zebra finch nomenclature. (B) Distributions of windowed *F*_ST_ values calculated in 50kb windows for each comparison. Distributions are shown for Autosomes and Chromosome Z separately.

In the early stage of the speciation continuum (South Island *vs.* Chatham Island and South Island *vs.* French Polynesia comparisons), the distributions of *F*_ST_ values calculated in 50kb windows for autosomes were highly skewed toward large values of *F*_ST_ (skew = 2.69 and 2.07 respectively) (Figure 3B) and characterized by extreme L-shaped distributions with divergence limited to few 50kb windows (Figure 4B). Over longer divergence timeframes distributional skew was reduced, with the lowest levels of skew (skew = 1.41) observed for the late stage – no gene flow comparison (Mainland *vs.* Lord Howe Island) (Figure 3B). The accumulation of genome-wide divergence over longer divergence timeframes is clearly visible in Figure 4A. When matched for divergence timeframe, the *F*_ST_ distributions of populations diverging under the influence of gene flow showed higher levels of skew (more localized divergence) than populations diverging in the absence of gene flow – this difference was significant for the two late-stage comparisons (*P* < 0.001). Although not all pairwise differences were significant, recently diverging populations in most cases had significantly higher skew for autosomes than late stage diverging populations (Table 2). When treating each autosomal chromosome as an independent unit, more chromosomes showed significantly reduced *F*_ST_ distributional skew than increased skew when diverging under gene flow, during both early and late stage divergence (Table S2).

**Table 2:** P-values for pairwise comparisons of *F*_ST_ distributional skew tested using a randomization test. Below diagonal = autosomes only; above diagonal = chromosome Z only

	SI *vs.* CI	SI *vs.* FP	ML *vs.* HI	GT *vs.* LF	ML *vs.* LH
**SI *vs.* CI**	—	0.688	0.967	0.732	0.040
**SI *vs.* FP**	0.447	—	0.593	0.885	0.005
**ML *vs.* HI**	<0.001	<0.001	—	0.226	0.031
**GT *vs.* LF**	<0.001	0.225	0.062	—	0.004
**ML *vs.* LH**	<0.001	<0.001	0.060	<0.001	—

Distributional skew of chromosome Z was lowest for the Mainland *vs.* Lord Howe Island (late stage - no gene flow) comparison, meaning that divergence was more widespread for this comparison (Figure 4A). This difference in skew was significant when compared to all other diverging population comparisons (all *P*-values <0.05, Table 2). Unlike for autosomes, there were no significant differences in chromosome Z distributional skew between South Island *vs.* Chatham Island, South Island *vs.* French Polynesia, Mainland *vs.* Heron Island, and Grande Terre *vs.* Lifou comparisons (Table 2).

### Dynamics of genomic islands and genomic valleys

**Figure 5:**
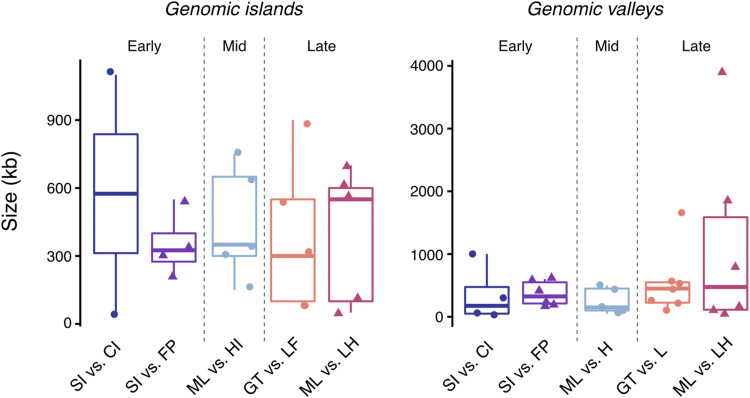
Size (kb) of genomic islands and genomic valleys identified for each population comparison. Populations diverging with gene flow are indicated by circles and populations diverging in isolation by triangles.

Genomic islands were identified in all population comparisons, including populations diverging with gene flow (South Island *vs.* Chatham Island, Mainland *vs.* Heron Island, and Grande Terre *vs.* Lifou) as well as populations diverging in the absence of gene flow (South Island *vs.* French Polynesia and Mainland *vs.* Lord Howe Island) (Figure 4A). Genomic island size – although variable (mean size ranged from 350 kb to 575 kb, see Table 3 and Figure 5) – did not differ significantly between population comparisons (Kruskal-Wallis: ${\text{χ}}_{4}^{2}$ = 0.44, *P* = 0.98). This finding was robust to different window sizes, with no significant difference in genomic island size observed when identifying genomic islands using a 5kb window (Kruskal-Wallis: ${\text{χ}}_{4}^{2}$ = 7.897, *P* = 0.095) or 100kb window (Kruskal-Wallis: ${\text{χ}}_{4}^{2}$ = 1.43, *P* = 0.84) (see Figure S1). We identified one region of the genome in which a genomic island of divergence occurred at the same location across multiple population comparisons. This genomic island, which was located on chromosome 5 and occurred in both late stage comparisons, was largest for the Mainland *vs.* Lord Howe Island comparison (Late stage – No gene flow) (Table 4). In most instances, absolute divergence (*d*_xy_) within individual genomic islands was not significantly different from background chromosomal levels. However, genomic islands with significantly elevated *d*_xy_ were identified in small numbers, as were genomic islands with below background level *d*_xy_ (Figure 4A).

**Table 3:** Summary statistics of genomic islands and genomic valleys identified for each population comparison

Divergence timeframe	Mode of divergence	Comparison	No. identified	Mean size (kb)	Max size (kb)	Mean *F*_ST_	Mean *d*_xy_
**Genomic islands**							
Early stage	Gene flow	SI *vs.* CI	2	575	1,100	0.031	0.003
Early stage	No-gene flow	SI *vs.* FP	4	350	550	0.038	0.005
Mid stage	Gene flow	ML *vs.* HI	5	440	750	0.147	0.005
Late stage	Gene flow	GT *vs.* LF	5	390	900	0.233	0.002
Late stage	No-gene flow	ML *vs.* LH	5	400	700	0.153	0.006
**Genomic valleys**							
Early stage	Gene flow	SI *vs.* CI	4	350	1,000	0.001	0.005
Early stage	No-gene flow	SI *vs.* FP	6	367	600	0.005	0.003
Mid stage	Gene flow	ML *vs.* HI	5	250	500	0.040	0.004
Late stage	Gene flow	GT *vs.* LF	7	536	1,650	0.019	0.002
Late stage	No-gene flow	ML *vs.* LH	6	1,142	3,900	0.059	0.003

**Table 4:** Size of genomic islands and genomic valleys occurring at the same location across multiple population comparisons

Divergence timeframe	Mode of divergence	Comparison	Genomic island size (kb)	Genomic valley size (kb)	
			5	1	7
Early stage	Gene flow	SI *vs.* CI	—	—	—
Early stage	No-gene flow	SI *vs.* FP	—	—	—
Mid stage	Gene flow	ML *vs.* HI	—	—	—
Late stage	Gene flow	GT *vs.* LF	100	250	450
Late stage	No-gene flow	ML *vs.* LH	550	1,850	3,900

Genomic valleys were identified in all diverging population comparisons (Figure 4A). Their size – although variable (mean size ranged from 250 kb to 1,142 kb, see Table 3 and Figure 5) – did not differ significantly between population comparisons (Kruskal-Wallis: ${\text{χ}}_{4}^{2}$ = 2.45, *P* = 0.65). Although significant differences in genomic valley size were observed when identifying genomic valleys using a 5kb window (Kruskal-Wallis: ${\text{χ}}_{4}^{2}$ = 14.95, *P* < 0.01) and 100kb window (Kruskal-Wallis: ${\text{χ}}_{4}^{2}$ = 9.61, *P* < 0.05) (see Figure S1), Dunn’s post-hoc test indicated that this difference was limited to few pairwise comparisons and followed no specific pattern in terms of gene flow scenario or divergence timeframe (Table S3). We identified two regions of the genome (located on chromosomes 1 and 7) in which a genomic valley occurred across multiple population comparisons (Table 4). Both shared genomic valleys were identified in the late stage comparisons and were largest for the comparison thought to be diverging in the absence of gene flow (Mainland *vs.* Lord Howe Island). In most instances, absolute divergence (*d*_xy_) within individual genomic valleys was not significantly different from background chromosomal levels. However, genomic valleys with significantly decreased *d*_xy_ were also frequently identified (Figure 4A). A single genomic valley with significantly elevated *d*_xy_ was identified for the South Island *vs.* Chatham Island comparison (Early stage – Gene flow).

### Correspondence of genomic islands and valleys to the position of outlier SNPs

**Figure 6:**
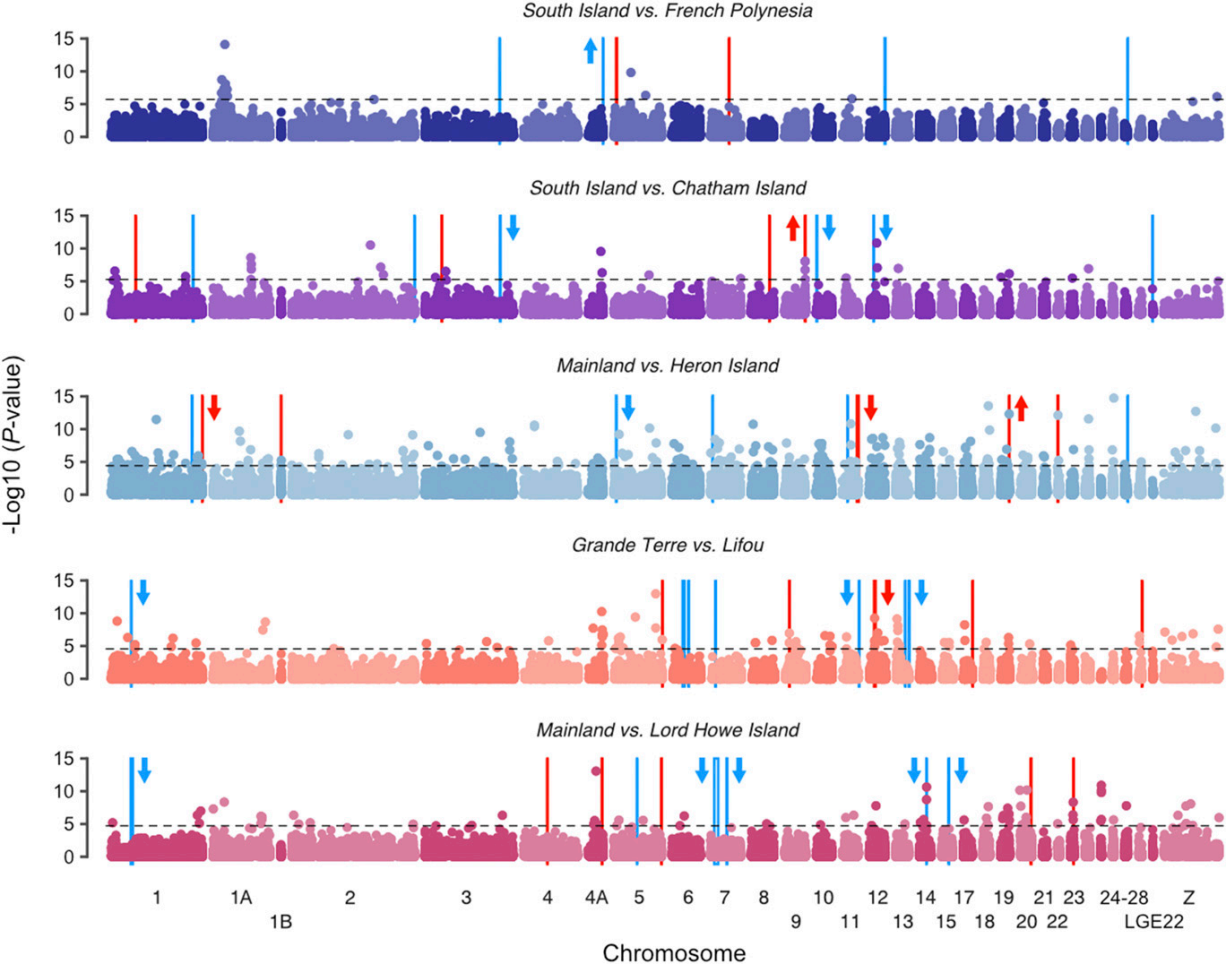
Manhattan plot of negative log10 (*P*-values) estimated using PCAdapt. Dashed line indicates *P*-value threshold above which SNPs are determined outliers (FDR = 0.01). 50kb windows with elevated differentiation (genomic islands) are highlighted in red and regions with low differentiation (genomic valleys) are highlighted in blue. Genomic islands and genomic valleys are based on *F*_ST_ and locations are the same as those shown in Figure 4. Arrows beside genomic islands/valleys indicate if *d*_xy_ within these regions was significantly elevated (upwards pointing arrows) or decreased (downward pointing arrows) compared to chromosomal background levels. Significance determined using Wilcoxon signed-rank tests. Chromosomes are numbered according to the zebra finch nomenclature.

*PCAdapt* identified between 14 and 235 outlier SNPs in each population comparison (Table 5 and Figure 6). For the South Island *vs.* Chatham Island, Mainland *vs.* Heron Island, and Grande Terre *vs.* Lifou comparisons the position of four outlier SNPs fell within genomic islands of divergence. A single outlier SNP was identified as occurring within a genomic valley for the Mainland *vs.* Lord Howe Island comparison.

**Table 5:** Outlier SNP summaries. Number of outlier SNPs identified using *PCAdapt* and the number located within genomic islands and genomic valleys per population comparison

Divergence timeframe	Mode of divergence	Comparison	No. outliers identified	No. outliers in genomic islands	No. outliers in genomic valleys
Early stage	Gene flow	SI *vs.* CI	14	0	0
Early stage	No-gene flow	SI *vs.* FP	28	4	0
Mid stage	Gene flow	ML *vs.* HI	235	4	0
Late stage	Gene flow	GT *vs.* LF	89	4	0
Late stage	No-gene flow	ML *vs.* LH	76	0	1

### Candidate gene analysis

A review of the literature identified 22 candidate genes associated with body and/or beak size differences in birds, for which we had sequence data or occurred within the 50kb windows used when summarizing divergence statistics (Table 6). The location of candidate genes did not correspond to the position of any genomic islands or genomic valleys identified. However, for the South Island *vs.* French Polynesia (Early stage – No gene flow) and Mainland *vs.* Heron Island (Mid stage – Gene flow) comparisons an outlier SNP was located within *BMPR1A* (Figure 6 and Table 6). This was the only candidate gene to contain outlier SNPs.

**Table 6:** Patterns of differentiation in genes linked to body/bill size differentiation in birds

Gene	Chromosome	Source	Associated with bill or body size?	Within genomic island?	Mapped to by outlier SNPs?
*ALX1*	1A	*^a^*	Bill	N	N
*ALX4*	5	*^b^*	Bill	N	N
*BMP4*	5	*^c^*,*^d^*	Bill	N	N
*BMPR1A*	6	*^b^*	Bill	N	Y (SI *vs.* FP and ML *vs.* HI)
*CACNA1G*	18	*^e^*	Bill	N	N
*COL17A1*	6	*^f^*	Body	N	N
*COL4A5*	4A	*^b^*	Bill	N	N
*COL6A3*	7	*^f^*	Body	N	N
*FGF10*	Z	*^a^*	Bill	N	N
*FOXC1*	2	*^a^*	Bill	N	N
*GSC*	5	*^a^*	Bill	N	N
*HMGA2*	1A	*^g^*	Bill	N	N
*IGF1*	1A	**^h^**	Bill	N	N
*INHBA*	2	*^b^*	Bill	N	N
*ITPR2*	1A	*^f^*	Body	N	N
*ITPR3*	26	*^f^*	Body	N	N
*NELL1*	5	*^b^*	Bill	N	N
*RDH14*	3	*^a^*	Bill	N	N
*SATB2*	7	*^b^*	Bill	N	N
*SIX2*	3	*^b^*	Bill	N	N
*TRPS1*	2	*^b^*	Bill	N	N
*VPS13B*	2	*^a^*,*^b^*	Bill	N	N

a Lamichhaney *et al.* (2015).

b Bosse *et al.* (2017).

c Abzhanov *et al.* (2004).

d Campàs *et al.* (2010).

e Cheng *et al.* (2017).

f Cornetti *et al.* (2015).

g Lamichhaney *et al.* (2016).

h vonHoldt *et al.* (2018).

### Simulations of neutral divergence

**Figure 7:**
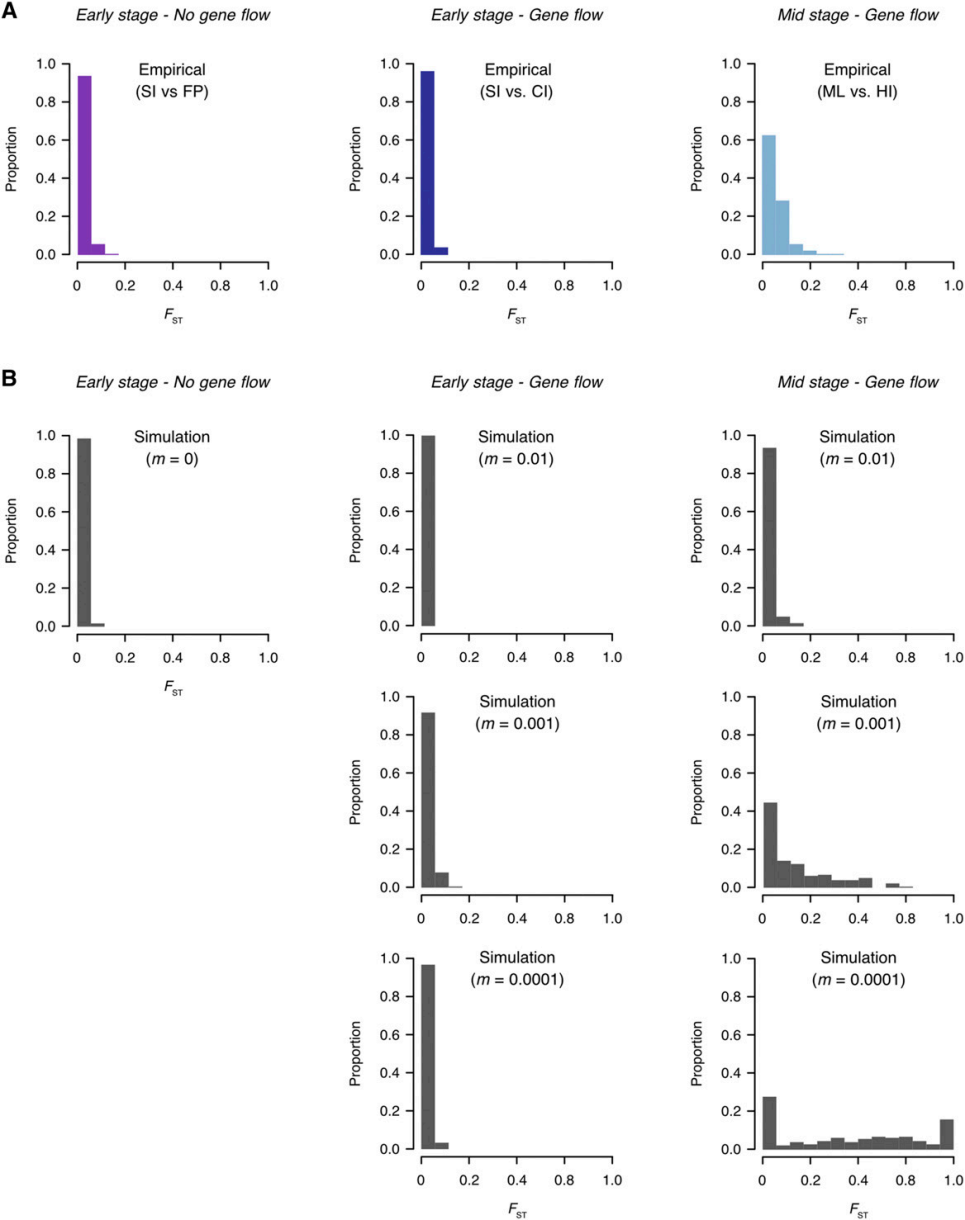
Comparison of observed and simulated *F*_ST_ distributions. (A) Frequency distributions of pairwise differentiation (*F*_ST_) for empirical data (chromosome 5 only). (B) Frequency distributions of pairwise differentiation (*F*_ST_) for simulated data. The *F*_ST_ values are calculated in 500kb windows. Simulations were conducted using a recombination rate of 3 cM/Mb (the distance between loci for which the expected average number of intervening chromosomal crossovers in a single generation is 0.01). Simulations of divergence with gene flow were conducted under different migration rates (*m* = 0.01, *m* = 0.001, and *m* = 0.0001). Simulation timeframes matched that for each empirical comparison (Early stage - no gene flow: 27 generations; Early stage - gene flow: 47 generations; Mid stage - gene flow: 1,000 generations).

**Figure 8:**
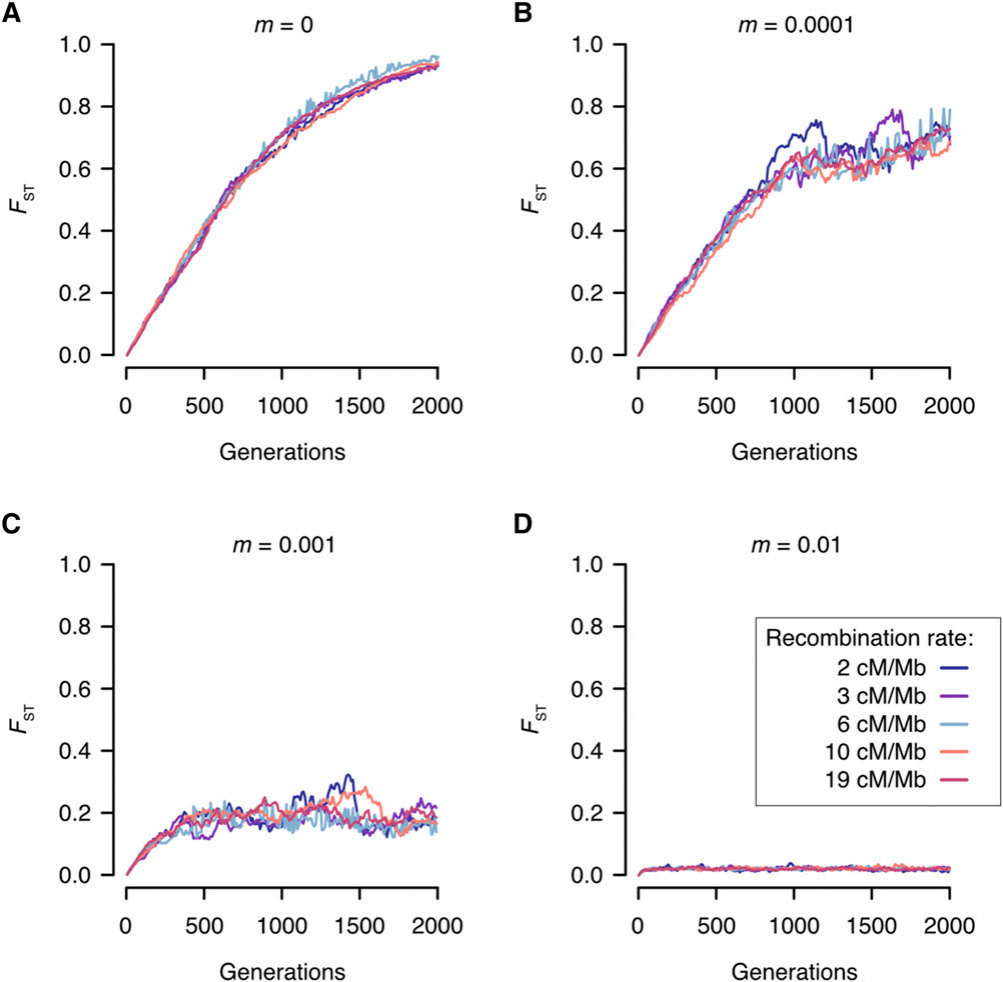
Expected *F*_ST_ values for 2,000 generations of simulated neutral divergence. (A) During divergence without gene flow (*m* = 0) *F*_ST_ rapidly increases, achieving fixation at 2,000 generations under all recombination rates; (B) During divergence with low levels of migration (*m* = 0.0001) *F*_ST_ increases rapidly, approaching fixation at 2,000 generations for all recombination rates; (C) During divergence with intermediate levels of migration (*m* = 0.001) *F*_ST_ increases rapidly during the initial ∼300 generations, after which it continues to fluctuate around 0.2 with some variation between recombination rates; and (D) During divergence with high levels of gene flow (m = 0.01) low levels of *F*_ST_ are maintained across all 2,000 generations of divergence.

Simulations of neutral divergence, using an individual based model, were able to capture the transition from localized to more genome-wide levels of divergence, in which *F*_ST_ distributions were characterized by low values of *F*_ST_ during the early stages of divergence, shifting toward higher *F*_ST_ values over longer divergence timeframes (Figure 7). Simulating divergence under various levels of gene flow (*m* = 0, *m* = 0.0001, *m* = 0.001, *m* = 0.01) showed that the accumulation of divergence slowed under increasing levels of gene flow (Figure 8).

## Discussion

We take advantage of an exceptional natural system where gene flow scenarios and divergence timescales are either known *a priori* or can be reasonably presumed, to reveal key insights into how divergence accumulates at the genomic level. Our results provide an empirical characterization of the change in the distribution of genomic divergence using a proxy for the speciation continuum. The pattern of decreasing skew in the distribution of genomic divergence is consistent with the intuitive explanation of how heterogeneity originates, and divergence accumulates, *i.e.*, divergence at a small number of selected loci followed by divergence hitchhiking. However closer inspection of the genomic landscape provides equivocal support for this particular mechanism. Genomic islands were rarely associated with SNPs putatively under selection and genomic islands did not widen as expected under the divergence hitchhiking model of speciation. Genomic valleys of similarity were also a common feature of the heterogeneous genomic landscape, but we did not find clear evidence to attribute these to selective processes. Furthermore, by simulating divergence under a neutral model we were able to capture the transition from localized to more genome-wide levels of divergence. The transition from localized divergence (highly skewed distribution) to more genome-wide levels of divergence (a more even distribution of genetic differentiation) therefore appears to occur largely outside of the context of genomic islands and is not dependent of selection, raising questions about the divergence hitchhiking model of speciation, where divergence is initiated through selection of a small number of genes (presumably of large effect), genomic islands form around them, and divergence hitchhiking completes genome-wide divergence.

### The accumulation of divergence across the genome

Our results demonstrate how divergence accumulates across the genome as populations move along the speciation continuum. *Zosterops lateralis* populations at the earliest stages of divergence followed extreme L-shaped *F*_ST_ frequency distributions where divergence was limited to few loci, with a reduction of skew in *F*_ST_ values as divergence became more wide-spread at later stages of the speciation continuum. This shift in distribution occurred in both the presence and absence of gene flow. We are aware of only two other studies where similar comparisons could be made: *Heliconius* butterflies (Martin *et al.* 2013) and *Ficedula* flycatchers (Burri *et al.* 2015). Unlike Burri *et al.* (2015), we did not observe a shift toward an extreme left-skewed distribution where the majority of SNPs reach fixation. This is likely due to the inclusion of comparisons between flycatcher species with divergence times of millions of years, whereas our within-species divergence timeframes were too short for an extreme left-skewed distribution to be reached.

On the whole, the observed distributions of genomic differences across the genome fit with expectations for how genomic divergence accumulates across the speciation continuum (Nosil and Feder 2012; Seehausen *et al.* 2014). However, our simulations demonstrated that the shift from localized to more genome-wide levels of divergence could be achieved assuming a neutral model of molecular evolution. This finding was robust to varying levels of recombination, which is interesting given the emphasis on linkage disequilibrium in driving localized and ultimately genome-wide divergence (Seehausen *et al.* 2014). One important difference, however, was that our simulations suggest that the transition from localized to more genome-wide levels of divergence occurs at a faster rate than is observed empirically - under allopatry and low-level gene flow *F*_ST_ approached fixation by 2,000 generations. This suggests that some other mechanism, such as frequency-dependent selection, may be maintaining genetic variation at individual SNPs. However, our simulation framework only considered diverging population consisting of 400 individuals each, whereas in reality many of the diverging populations have much larger population sizes. As the potential for drift is greatest when population size is small (Wright 1931), this discrepancy in the pace of divergence may reflect differences in population size not captured in our simulation framework.

When matched for divergence timeframe, the pace at which divergence accumulated across the genome was moderated by gene flow. This was manifested as higher distributional skew of autosomal *F*_ST_ values for silvereye populations thought to be diverging under gene flow compared to populations thought to be diverging in genetic isolation. Martin *et al.* (2013) found that genomic divergence was more widespread for races and species of *Heliconius* butterflies diverging in geographic allopatry than for those diverging in parapatry and sympatry, suggesting an underlying role for gene flow in slowing the accumulation of genomic divergence. We provide a further empirical characterization of this difference in the accumulation of genomic divergence. While we can be certain that the Mainland and Heron Island populations have been diverging under the effects of gene flow (the mainland subspecies is frequently observed on Heron Island) and that the South Island and French Polynesian populations have been diverging under genetic isolation (the French Polynesian population is the product of a single human-mediated introduction well beyond the natural distribution limit for this species) it is important to state that there is some uncertainty as to the gene flow status of the other comparisons used in our study. However, with that said, the moderating effect of gene flow was also apparent when simulating divergence.

Given that the silvereye population in French Polynesia was the product of a human introduction and therefore likely had lower effective founder size than natural colonisations involving large expanding populations of silvereyes (Sendell-Price *et al.* 2020), we cannot rule out that the more wide-spread genomic divergence observed for the South Island *vs.* French Polynesia population comparison (when compared to other early stage comparison: South Island *vs.* Chatham Island) is the product of a potential severe population bottleneck during introduction. The potential for population founding to accelerate the accumulation of genomic differences has been highlighted previously (Ravinet *et al.* 2017), and in the silvereye system could be addressed through genomic comparisons of a number of other populations with varying intensities of population bottlenecks (Estoup and Clegg 2003).

Divergence of silvereye chromosome Z was elevated beyond that observed for autosomes, as seen in a number of other avian divergence studies (Storchová *et al.* 2010; Sætre and Sæether 2010; Ruegg *et al.* 2014; Oyler-McCance *et al.* 2015). Sex chromosomes are thought to contain a large number of genes related to sexual selection and reproductive isolation, and as such have been proposed to play a disproportionately large role in speciation (Dobzhansky 1974; Coyne 1985; Ellegren 2009; Charlesworth *et al.* 2017). Despite this, no genomic islands, and only a handful of significant outlier SNP, were identified within chromosome Z. Instead, the elevated levels of divergence may be partly explained by the lower effective population size of the Z chromosome (3/4 of an autosome), amplifying the effects of genetic drift (Mank *et al.* 2010).

### The dynamics of genomic islands of divergence and valleys of similarity

Our results run counter to several patterns expected from the divergence hitchhiking model of speciation. First, genomic islands of divergence were seen in similar frequency in populations diverging both with and without gene flow. This adds to a number of empirical studies in natural systems where genomic islands have been observed in populations that are, or are assumed to be, genetically isolated (Renaut *et al.* 2013; Marques *et al.* 2016; Han *et al.* 2017; Van Doren *et al.* 2017; Zhang *et al.* 2017). Second, genomic islands were not wider on average for older divergence timeframes, which does not align with the prediction that genomic islands expand with time as linkage disequilibrium facilitates divergence of neutral and weakly selected loci via divergence hitchhiking (Smadja *et al.* 2008; Via and West 2008; Via 2012). A small number of recent studies have also failed to find a positive association between divergence time and average genomic island size (*Timema* stick insects (Riesch *et al.* 2017); Darwin’s finches (Han *et al.* 2017); and Yellow-rumped warblers (Toews *et al.* 2016)). Taken together, growth of genomic islands may not be pervasive in the generation of genome-wide divergence, with the accumulation of divergence in our example instead taking place largely outside of the context of genomic islands. However, given that we have identified relatively few genomic islands we may lack the statistical power needed to detect differences in genomic island size across different divergence timeframes and gene flow scenarios. We therefore caution readers against over-interpreting this primarily negative result.

Associating average genomic island size with divergence time may be too coarse an approach, and comparisons of genomic islands at specific genomic locations may be a better method to understand how time and gene flow shape growth of genomic islands. In our case, only one genomic island was located at overlapping genomic positions across population comparisons, however this genomic island did not occur across different divergence timeframes. Whole genome analysis would likely provide more shared genomic islands, but currently we lack support for the idea that expansion of genomic islands of divergence at specific locations over time occurs.

Contrary to the expectation that genomic islands of divergence contain loci under strong divergent selection (Wu 2001), we find that genomic islands regularly occur in regions of the *Z. lateralis* genome where outlier SNPs putatively under directional selection were not identified. A similar result was reported in diverging Swainson’s thrush *Catharus ustulatus*) subspecies (Ruegg *et al.* 2014). Hence, it appears that genomic islands of divergence are not always seeded by directional selection, but whether this is frequently the case requires a broader number of empirical examples. The formation of genomic islands without obvious evidence of directional selection highlights the need for alternative explanations for their formation (Turner *et al.* 2005; Hohenlohe *et al.* 2010; Feder *et al.* 2013). One such explanation is that genomic islands of divergence may arise in regions of low recombination such as centromeres (Carneiro *et al.* 2009; Renaut *et al.* 2013; Burri *et al.* 2015). However, lack of recombination and karyotype maps for silvereyes at present prevents formal testing of this hypothesis here. In addition to variation in recombination rate, other genomic features that may contribute to the formation of genomic islands include variation in gene density and variation in mutation rate along the genome (Ravinet *et al.* 2017). Whole genome sequencing would help to address the contribution of these genomic features.

A comparison of absolute and relative measures of divergence (*e.g.*, *d*_xy_ and *F*_ST_ respectively) can provide further insight into the formation of genomic islands (Nachman and Payseur 2012; Han *et al.* 2017; Delmore *et al.* 2018). Unlike *F*_ST_, which is consistently elevated around loci under directional selection, *d*_xy_ may be elevated, reduced or unchanged. Under divergence with gene flow, genomic islands with elevated measures of both relative and absolute divergence are expected to form around regions where gene flow is disadvantageous, such as around regions containing variants involved in local adaptation or reproductive isolation (Han *et al.* 2017). We observed such a pattern in only one of the three comparisons diverging with gene flow. In the absence of gene flow, selective sweeps, which convert between-population variation into fixed differences, are expected to produce regions of elevated *F*_ST_ but not *d*_xy_ (Nachman and Payseur 2012; Cruickshank and Hahn 2014). Such islands were frequently observed across all comparisons, suggesting that genomic islands arise frequently within regions where gene flow is absent. Genomic islands (based on *F*_ST_) but with reduced *d*_xy_, were also frequently observed. Such a pattern is thought to be caused by background selection and recurrent selective sweeps, both of which result in locally reduced levels of genetic variation (Seehausen *et al.* 2014; Wolf and Ellegren 2016; Ma *et al.* 2017).

Genomic valleys play an important role in shaping the genomic landscape across the speciation continuum, occurring across all population comparisons. In particular, their occurrence during the late stage of divergence may aid maintenance of heterogeneity by slowing the approach to genome-wide divergence at some genomic regions. Should genomic valleys remain present in the very advanced stages of divergence - *i.e.*, comparisons at the species level - these regions likely contribute to the formation of the tail of extreme values that characterizes the left skewed distributions observed during the late stage of divergence such as those observed between diverging *Ficedula* flycatcher species by Burri *et al.* (2015). Comparisons between *Zosterops* species are needed to confirm this expectation.

A comparison of the nature of genomic valleys across the speciation continuum and gene flow contexts allows us to comment on mechanisms proposed to generate genomic valleys. Although genomic valleys frequently showed a pattern of reduced *F*_ST_ and *d*_xy_, as would be expected if genomic valleys form at loci under parallel selection (Roesti *et al.* 2014) or purifying selection (Cvijović *et al.* 2018), in few cases did the position of an outlier SNP correspond to the position of a genomic valleys. As such, at present we have limited evidence to confirm that genomic valleys frequently arise due to effects of parallel or purifying selection. Second, as the size of genomic valleys was found to be stable over time, we do not find support that genomic valleys are solely the product of incomplete lineage sorting at neutral loci, as under this mechanism genomic valleys would be expected to become smaller as shared ancestral variation breaks down over time (Stölting *et al.* 2013).

### Repeated evolutionary patterns

While we found no evidence that candidate genes thought to be associated with body and/or bill size differences in passerines were concentrated within genomic islands, we did find evidence that one such gene may be implicated in the repeated evolutionary pattern seen in island silvereyes. Bone morphogenetic protein receptor, type 1A (*BMPR1A*) was identified as containing outlier SNPs potentially under selection in both the South Island *vs.* French Polynesia and Mainland *vs.* Heron Island comparisons. This gene is associated with the ontology term “palate development” [Gene Ontology (GO) 0060021] and has been previously identified as a candidate gene underlying bill length variation in great tits (*Parus major*) (Bosse *et al.* 2017). Interestingly, both French Polynesia and Heron Island populations have increased bill sizes (and overall body size) when compared to their respective source populations (Clegg *et al.* 2010; Sendell-Price *et al.* 2020). A further gene within the Bone Morphogenetic Protein (BMP) pathway, *BMP4*, has been strongly associated with bill shape variation in Darwin’s finches (genus: *Geospiza*) (Abzhanov *et al.* 2004).

## Conclusion

While the goal of identifying unambiguous signatures of particular evolutionary processes in patterns of genomic divergence remains elusive, the addition of empirical studies of well-characterized systems provides valuable insight into the nature of divergence across the speciation continuum. In the silvereye system, gene flow is clearly important in shaping genome-wide divergence. While these results provide an excellent baseline for inferring the role of gene flow in other silvereye populations, equivalent calibrations would likely be needed on a species-by-species basis for the role of gene flow in a population of unknown history to be assessed. Genome-wide divergence in silvereyes does not hinge on the formation and growth of genomic islands. This is at odds with the intuitive and until recently, frequently invoked verbal model describing the accumulation of genomic divergence during the speciation continuum. Instead, differences are spread in a remarkably even way across the genome. The joint empirical and theoretical approach we present offers a potentially powerful tool to test a range of hypotheses about the mechanisms that underlie genomic speciation.
